# Depressive Symptoms and the Risk of Ischemic Stroke in the Elderly—Influence of Age and Sex

**DOI:** 10.1371/journal.pone.0050803

**Published:** 2012-11-30

**Authors:** Christian L. Seifert, Holger Poppert, Dirk Sander, Regina Feurer, Thorleif Etgen, Karl-Heinz Ander, Klaus Pürner, Monika Brönner, Dominik Sepp, Victoria Kehl, Hans Förstl, Horst Bickel

**Affiliations:** 1 Department of Neurology, Klinikum rechts der Isar, Technische Universität München, Germany; 2 Department of Psychiatry and Psychotherapy, Technische Universität München, Germany; 3 Department of Neurology, Kliniken Südostbayern – Klinikum Traunstein, Germany; 4 Department of Neurology, Benedictus Hospital, Tutzing, Germany; 5 Institute of Medical Statistics and Epidemiology, Technische Universität München, Germany; 6 INVADE Study Group, Baldham, Germany; University of Regensburg, Germany

## Abstract

Although a relationship between depression and cardiovascular events has been suggested, past study results regarding the risk of stroke in relation to depression by subgroups are ambiguous. The aim of this study was to investigate the influence of depressive symptoms on risk of incident ischemic stroke in elderly according to age and sex. This prospective cohort study followed up 3852 subjects older than 55 years. Baseline depressive symptoms were defined by a score ≥5 on the Geriatric Depression Scale or antidepressant intake. The outcome measure was incident ischemic stroke within 6 years of follow-up. Multivariate Cox-proportional hazard models as well as cumulative survival analyses were computed. A total of 156 ischemic strokes occurred during the study period (24 strokes in the age-group<65 years and 132 strokes in the age-group≥65 years). The distribution of strokes in sex-subgroups was 4.5% in men and 3.7% in women. The multivariate analysis showed an elevated stroke risk (Hazard Ratio (HR): 2.84, 95% CI 1.11–7.29, p = 0.030) in subjects from 55 to 64 years with depressive symptoms at baseline but not in subjects older than 65 years. In the multivariate analysis according to sex the risk was increased in women (HR: 1.62, 95% CI 1.02–2.57, P = 0.043) but not in men. The Cox-regression model for interaction showed a significant interaction between age and sex (HR: 3.24, 95% CI 1.21–8.69, P = 0.020). This study corroborates that depressive symptoms pose an important risk for ischemic stroke, which is particularly remarkable in women and patients younger than 65 years.

## Introduction

Stroke prevention requires the treatment of modifiable “classical risk factors” such as coronary heart disease (CHD), hypertension, cigarette smoking, diabetes mellitus, hyperlipidemia, obesity, atrial fibrillation, and physical inactivity as well as recently suggested or less well studied risk factors such as metabolic syndrome, excessive alcohol consumption, drug abuse, use of oral contraceptives, sleep-disordered breathing, migraine, hyperhomocysteinemia, elevated lipoprotein(a), hypercoagulability, inflammation, and infection [1]. Although affective disorders, to date, have not been formally established as independent risk factor for stroke, attention to this potential risk factor has continued to increase in the past decade [2]. The lifetime incidence of depression has been estimated at more than 16% in the general population [3] and an association between depression and medical diseases has been shown for diabetes [4], cardiovascular disease [5], [6], [7], [8], and hypertension [9]. A link between depression and the incidence of stroke has been strengthened by two recent meta-analyses [2], [10], but findings in elderly subgroups by age and sex are still partly ambiguous [11] and separate risk estimations for them are rarely available. Furthermore the studies included in the meta-analyses used different endpoints as some studies included intracerebral hemorrhage and transitory ischemic attacks (TIA) [2], [10].

Several prospective studies have investigated the relation between depressive symptoms and the incidence of stroke. However, studies using depression as a predictor have yielded mixed results. Whereas a recent large population-based study (n = 80 574 women aged 54 to 79 years, the Nurses’ Health Study) showed an increased stroke risk in women with a previous diagnosis of depression [12], other studies found no clear evidence of depression being a significant risk factor for cerebrovascular diseases [13], [14] or an increased risk only for patients younger than 65 years [11]. Furthermore sex differences in the clinical course and incidence of depression have been repeatedly shown [15], [16].

**Table 1:** Univariate Cox-PH Model for the Risk of Stroke in Patients With and Without Depression (GDS ≥5)/Antidepressant Medication.

	Age <65				Age ≥65				All			
	Events/N	HR	95% CI	*P*-value (Wald)	Events/N	HR	95% CI	*P*-value (Wald)	Events/N	HR	95% CI	*P*-value (Wald)
GDS ≥5	24/1658	2.41	0.96 to 6.06	0.063	132/2185	1.51	0.99 to 2.30	0.057	156/3852	1.72	1.17 to 2.53	0.006
Antidepressant	24/1658	1.26	0.30 to 5.34	0.758	132/2185	1.07	0. 58 to 1.98	0.841	156/3852	1.15	0.65 to 2.02	0.639
GDS ≥5 or antidepressant	24/1658	2.17	0.90 to 5.23	0.085	132/2185	1.55	1.05 to 2.27	0.026	156/3852	1.74	1.23 to 2.48	0.002

**Table 2:** Multivariate Cox-PH Model for the Risk of ischemic Stroke in Subjects With and Without Depression (GDS ≥5)/Antidepressant Medication, Adjusted for Age and Sex.

	Age <65				Age ≥65				All			
	Events/N	HR	95% CI	*P*-value (Wald)	Events/N	HR	95% CI	*P*-value (Wald)	Events/N	HR	95% CI	*P*-value (Wald)
GDS ≥5	24/1658	2.81	1.11 to 7.11	0.029	132/2185	1.30	0.84 to 1.99	0.239	156/3852	1.41	0.95 to 2.08	0.087
Antidepressant	24/1658	1.51	0.36 to 6.46	0.576	132/2185	1.08	0.58 to 2.01	0.809	156/3852	1.15	0.65 to 2.03	0.644
GDS ≥5 or antidepressant	24/1658	2.59	1.07 to 6.28	0.035	132/2185	1.41	0.96 to 2.08	0.083	156/3852	1.51	1.06 to 2.16	0.023

In the present study, we investigated an association between depression and incident ischemic stroke among elderly patients with adjustment for the established risk factors. Because the incidence and characteristics of both depression and stroke changes with age, we follow the example of the Framingham study and performed separate analyses for subjects younger than 65 years and for subjects aged 65 years or older [11] and moreover for men and women respectively.

## Materials and Methods

### Subjects

This study is based on data from the INVADE trial (intervention project on cerebrovascular diseases and dementia in the district of Ebersberg), a population-based longitudinal study of general-practice patients. The study population is made up of the inhabitants of the district of Ebersberg, Bavaria, Germany, who were born before 1946 and were members of the public health insurance AOK (Allgemeine Ortskrankenkasse). In Bavaria the AOK is the biggest public health insurance with a market share over 40%. At the beginning of the year 2001, all members were invited to participate [17], [18], [19]. During the baseline period (2001–2003), 3908 subjects accepted the invitation to participate. Ultimately, for n = 3852 complete data for both Geriatric Depression scale and antidepressant intake were available. From 533 subjects who dropped out within the study period, 477 dropped out because of death and only 56 (1.4% of the whole study population) because of other reasons, e.g. emigration or a change of health insurance. The median follow up time until either the occurrence of an event or the end of the study was 6.13 years.

**Table 3:** Multivariate Cox-PH Model for the Risk of Stroke in Subjects With and Without Depression (GDS ≥5)/Antidepressant Medication, Adjusted for Known Risk Factors*.

	Age <65				Age ≥65				All			
	Events/N	HR	95% CI	*P*-value (Wald)	Events/N	HR	95% CI	*P*-value (Wald)	Events/N	HR	95% CI	*P*-value (Wald)
GDS ≥5	24/1609	3.23	1.19 to 8.81	0.022	131/2110	1.09	0.70 to 1.69	0.715	155/3727	1.20	0.80 to 1.79	0.380
Antidepressant	24/1609	1.67	0.38 to 7.34	0.498	131/2110	0.89	0.47 to 1.68	0.712	155/3727	0.94	0.52 to 1.68	0.826
GDS ≥5 or antidepressant	24/1609	2.84	1.11 to 7.29	0.030	131/2110	1.20	0.80 to 1.79	0.380	155/3727	1.30	0.90 to 1.89	0.158

* Adjusted for age, sex, BMI, smoking, hypertension, diabetes, hyperlipidemia, previous myocardial infarction, previous TIA, previous stroke, history of atrial fibrillation, and physical activity.

HR, hazard ratio.

### Baseline Investigation

The baseline investigation was performed by 65 primary care physicians of the district of Ebersberg and included a standardized questionnaire, a physical examination, evaluation of several risk factors, medical and disease history, a 12-lead ECG, and an overnight fasting venous blood sample for laboratory analysis including serum glucose, lipids, and creatinine as well as high sensitivity C-reactive protein (hs-CRP). Information on medical history, current health status, cognitive status, mood disorders, previous cardiovascular risk factors and drug usage was obtained using a structured interview. A physical examination was carried out by the primary care physician including the following items: weight and height with calculation of body-mass index (BMI); hypertension (treatment with antihypertensive medication or documented blood pressure ≥140 mmHg systolic or ≥90 mmHg diastolic, measured in a standardized fashion); diabetes mellitus (treatment with antidiabetic drugs or overnight fasting serum glucose levels ≥126 mg/dL); hyperlipidemia (treatment with lipid-lowering medication or total cholesterol level ≥200 mg/dL or triglyceride ≥150 mg/dL); 6-Item Cognitive Impairment Test [20]; Barthel Idex [21]; Rankin Scale [21]; medication; physical activity; history of stroke (neurological deficit that persisted longer than 24 hours, evaluated by a neurologist); history of ischemic heart disease (documented by previous myocardial infarction or angina pectoris, bypass surgery, or >50% angiographic stenosis of ≥1 major coronary artery); smoking status (never, former, or current); alcohol consumption; and living facility. The GDS was included in a self-administered patient questionnaire. All patients were monitored under best medical treatment conditions during the study period; the treatment by the primary care physicians followed the actual national and international guidelines. All subjects gave written informed consent before entering the study. The study was approved by the local ethics committee of the Technische Universität München.

### Depressive Symptoms, Geriatric Depression Scale

Depressive symptoms were assessed using the Geriatric depression scale (GDS), which was developed to estimate depression especially in the elderly [22]. In this questionnaire patients are asked to respond with reference to how they felt over the previous week. We used the 15-item version [23]. The GDS was found to have a sensitivity of 92% and specificity of 89% when evaluated against diagnostic criteria [24]. We used the cut-off ≥5 in the GDS-15 which had a reported sensitivity for the detection of major depressive episode of >90% [25]. In addition, depressive symptoms were assumed if the general practitioner reported the prescription of any established antidepressant medication (selective serotonin reuptake inhibitors (SSRI), Tricyclic antidepressants (TCA), Monoamin-oxidase (MAO)-inhibitors, etc.).

### Clinical End Point

The subjects enrolled in the study were monitored for all ischemic strokes according to the ICD classification (International Statistical Classification of Diseases and Related Health Problems, World Health Organization) through a linkage of the study database with claims data of the health insurance company. In case of the occurrence of an ischemic stroke the ICD code - coded as diagnosis by the physicians in hospitals - were registered in the AOK database. As we used insurance claim data in terms of ICD codes (I63.- for ischemic stroke) reported by the hospitals, information on clinical endpoints were completely available for every participant except the ones who died or changed the insurance (1.4%) over the entire observation period.

**Table 4:** Sex-dependent multivariate Cox-PH Model for the Risk of Stroke in Subjects With and Without Depression (GDS ≥5)/Antidepressant Medication, Adjusted for Age.

	Women				Men			
	Events/N	HR	95% CI	*P*-value (Wald)	Events/N	HR	95% CI	*P*-value (Wald)
GDS ≥5	84/2264	1.37	0.83 to 2.28	0.220	72/1587	1.35	0.72 to 2.50	0.350
Antidepressant	84/2264	1.19	0.62 to 2.31	0.599	72/1587	0.97	0.31 to 3.09	0.963
GDS ≥5 or antidepressant	84/2264	1.75	1.11 to 2.75	0.015	72/1587	1.10	0.59 to 2.05	0.755

**Table 5:** Sex-dependent multivariate Cox-PH Model for the Risk of Stroke in Subjects With and Without Depression (GDS ≥5)/Antidepressant Medication, Adjusted for Known Risk Factors*.

	Women				Men			
	Events/N	HR	95% CI	*P*-value (Wald)	Events/N	HR	95% CI	*P*-value (Wald)
GDS ≥5	84/2202	1.34	0.80 to 2.26	0.273	71/1524	1.00	0.52 to 1.91	0.996
Antidepressant	84/2202	0.95	0.48 to 1.88	0.881	71/1524	0.73	0.22 to 2.44	0.614
GDS ≥5 or antidepressant	84/2202	1.62	1.02 to 2.57	0.043	71/1524	0.85	0.44 to 1.62	0.618

* Adjusted for age, sex, BMI, smoking, hypertension, diabetes, hyperlipidemia, previous myocardial infarction, previous TIA, previous stroke, history of atrial fibrillation, and physical activity.

HR, hazard ratio.

### Statistics

All patients from the INVADE project with a completed baseline GDS score were included in this study. The dichotomized GDS score (GDS ≥5) as well as current prescription of antidepressant medication was investigated together as well as separately as predictors of stroke. For the combined analysis with GDS ≥5 and prescription of antidepressants only subjects with complete data were included. The analysis was performed for two groups (age: 55 to 64 years and ≥65 years) according to the analysis of the Framingham study [11] and further for men and women separately. Kaplan–Meier survival estimates were used to visualize time to stroke. Crude and multivariate Cox proportional hazards (PH) models were computed within each age and sex group. In multivariate models the associations between depressive symptoms and the clinical endpoints were adjusted for age and sex only as well as for age, sex, BMI, smoking, hypertension, diabetes, hyperlipidemia, physical activity, previous myocardial infarction, previous TIA, previous stroke, and history of atrial fibrillation following reports about established risk factors [1], [26]. Survival curves were compared between sub-groups using the log-rank test. An interaction analysis was conducted using a Cox-PH regression model with the covariates age, sex and age x sex. All statistical tests were two-sided with significance level of 5%. All statistics were performed with IBM SPSS version 19.0 and 20.0 with guidance by an independent statistician (V.K.).

**Figure 1:**
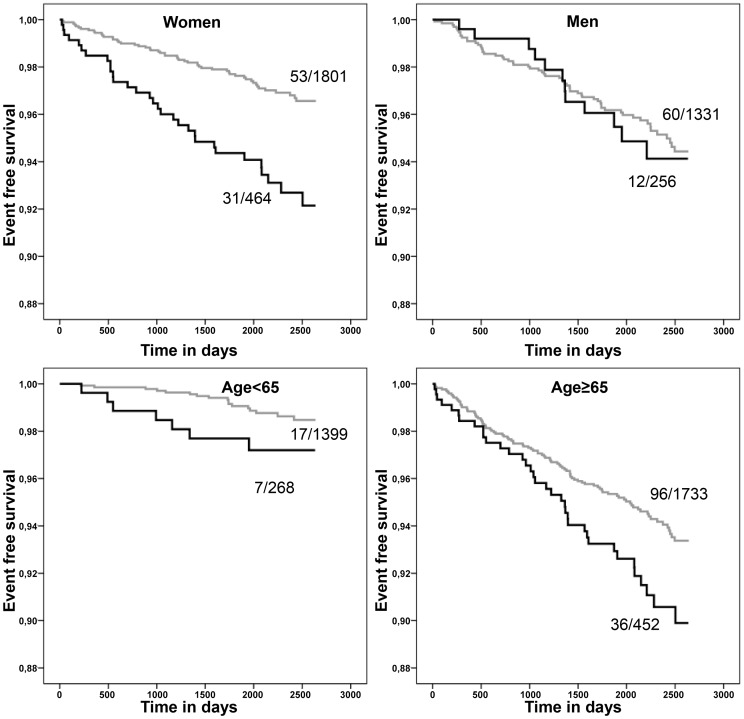
Kaplan–Meier survival plots for different age groups (<65, ≥65 years) and sex; timeframe between INVADE inclusion (day 0) and event free survival for ischemic stroke between the group taking antidepressants or GDS ≥5 and non-depressive group; for each plot the corresponding number of events and the size of the group is available at the end of the curve.

## Results

### Baseline Characteristics

Baseline characteristics of the study population in relation to age-group and depressive symptoms are summarized in Table S1. The mean baseline GDS scores differed between age groups and were 2.21 for subjects aged 55–64 years versus 2.61 for ≥65 years. In the age group<65 years (n = 1667), 16.1% had a GDS score ≥5 or antidepressant intake, whereas in the age group≥65 years (n = 2185), 20.7% had a GDS score ≥5 or antidepressant intake (p<0.001), indicating an age-dependent increase of depressive symptoms. The use of antidepressants did not differ between groups.

In the study population (N = 3852) 156 ischemic strokes occurred. In the age group<65 years (n = 1667) 24 ischemic strokes were observed, whereas in the group≥65 years (n = 2185), 132 strokes were recognized. In 1587 men and 2264 women the event rate was 4.5% in men (n = 72) and 3.7% (n = 84) in women.

### Uni- and Multivariate Analysis for Age-subgroups

The univariate Cox-PH analysis in the whole group indicated a significantly elevated risk for GDS only (hazard ratio (HR) 1.72, 95% confidence interval [CI] 1.17–2.53, P = 0.006) and the combined risk factor (GDS or antidepressant intake, HR 1.74, 95% CI 1.23–2.48, P = 0.002). The separate analysis for age groups showed a significant risk elevation (HR 1.55, 95% CI 1.05–2.27, P = 0.026) for ≥65 years and was non-significant (HR 2.17, 95% CI 0.90–5.23, P = 0.085) in <65 years for the combined risk factor (s. Table 1). However, use of antidepressant medication alone failed to reach statistical significance.

The multivariate Cox-PH model with adjustment for age and sex is presented in Table 2 while in comparison to the fully adjusted model presented in Table 3. The multivariate analysis with adjustment for age and sex only showed a significantly higher stroke risk in depressive patients below the age of 65 years (HR 2.59, 95% CI 1.07–6.28, *P* = 0.035, see Table 2) and also in the multivariate analysis with adjustment for the established risk factors (HR 2.84, 95% CI 1.11–7.29, P = 0.030, see Table 3) but not in the group of participants ≥65 years.

The multivariate analysis for GDS ≥5 only showed an HR of 2.81 for <65 years (95% CI 1.11–7.11, P = 0.029) adjusted for sex and age (Table 2) and a HR of 3.23 (95% CI 1.19–8.81, P = 0.022) adjusted for the known risk factors (Table 3). No significant association was seen in the group of subjects ≥65 years as well as for the whole group in the multivariate analysis adjusted for the known risk factors (see Table 3).

### Multivariate Analysis for Sex Subgroups (Tables 4 and 5)

The analyses for sex subgroups are presented in Tables 4 and 5. The multivariate Cox-PH model with adjustment for age showed a significantly higher stroke risk in depressive subjects only for women (HR 1.75, 95% CI 1.11–2.75, *P* = 0.015, see Table 4) and also in the multivariate analysis with adjustment for the established risk factors (HR 1.62, 95% CI 1.02–2.57, P = 0.043, see Table 5) but not in the group of men.

### Survival Analysis

Kaplan-Meier estimates for ischemic stroke were conducted by age and sex-subgroups (Fig. 1). In the female group there was a significant difference in cumulative stroke free survival when comparing subjects with and without depressive symptoms (P<0.001). This observation could not be confirmed in the male group (P = 0.733). When based on age subgroups there was a significant difference in the older group (age ≥65 years) between subjects with and without depressive symptoms (P = 0.025). As well, there was a similar trend identified in the younger group (P = 0.078). (s. Figure 1).

### Interaction Analysis in Relation to Stroke

The Cox-PH regression for the interaction of age x sex resulted a HR of 3.24 (95% CI 1.21–8.69) with p = 0 0.020. The results for age and sex in the regression model were p<0.001, HR 0.11 (95% CI 0.05–0.25) for age and p = 0.394, HR 1.16, (95% CI 0.82–1.65) for sex.

## Discussion

### Study Findings and Design

Apart from the individual and socioeconomic burdens of depression itself [27] this study contributes further evidence that depression alters the risk of ischemic stroke in elderly subgroups. The relative risk for developing an ischemic stroke was significantly elevated in our study population with baseline depressive symptoms by a nearly threefold risk in the younger group<65 years (HR 2.81) generally confirming the results of the Framingham study, which showed an risk elevation only in younger patients [11]. In comparison with earlier studies on depression and stroke [2], our result in the group younger than 65 years in the multivariate analysis appears to be relatively high. In addition one recent meta-analysis by Pan et al. [2] actually reported also an elevated risk in a stratified analysis also for younger patients <65 years (HR 1.77). Of note, only two previous original studies on depression and stroke have reported results separately by age sub-groups: the Framingham study investigated people younger than 65 years and those 65 years or older [11] and the Established Populations for Epidemiologic Studies of the Elderly (EPESE) examined an older population with subgroups 65–74, and 75 years or older [28]. It is known from epidemiological studies that an increase in depressive symptoms appears above the age of 65 years in men and women [29]. Comparing our results with the meta-analysis of Pan et al. [2] the novel finding is that the multivariate analysis separated for sex also showed an increased risk only in women (HR 1.75, P = 0.015) with depressive symptoms but not in men. In a recent meta-analysis by Dong et al [10] which investigated the same studies for the most part as Pan et al. [2] no such stratified analyses for sex had been performed. Considering these different results, the lack of risk elevation in depressed subjects ≥65 years in the multivariate analysis noted in our study (Tables 2 and 3) might be caused by a lower association in men. This could be partly explained by an overbalance of other risk factors in this sub-group, but might also be influenced by a dissimulation of depressive mood in older men. Our data support the approach of an analysis grouped for age and sex as a significant interaction between age and sex has been shown in our regression model. Besides, there are sex differences in late-life depression that are not restricted to sex-dependent characteristics and behavior but seem to be associated with mortality [30]. In conclusion it seems entirely reasonable to analyze age and sex subgroups separately, particularly with regard future therapy studies.

### Pathomechanisms and Antidepressant Treatment

Plausible causal mechanisms for an increased cardiovascular risk in depression have been put forward in the literature, such as the promotion of a mild inflammatory process induced by depression [31], triggering dysregulation of hormonal systems [32] and resulting greater platelet activation [33] and therefore causing atherosclerosis [34]. It can be argued that depression leads to mild inflammatory responses [35]. It has been revealed in population-based studies that antidepressant use can also be associated with elevated CRP levels [36], possibly leading to a systemic inflammation independently of the symptoms of mental illness. The exact role of antidepressant use is not yet clear, but some of these drugs are suspected to cause a mild increase of stroke risk with recently reported adjusted HRs between 1.05 (95% CI 0.95–1.17) for tricyclic antidepressants, 1.21 (95% CI 1.11–1.32) for selective serotonin reuptake inhibitors, and 1.44 (95% CI 1.24­1.67) for other antidepressants for other antidepressants [37]. It must be mentioned, that the risk caused by antidepressants could not easily been separated from the risk caused by depression. Furthermore, despite the results of our multivariate analysis, it may be argued that subjects with depressive symptoms had more vascular risk factors and altered risk behaviors particularly physical inactivity as shown for coronary heart disease [38]. From a clinical point of view, a possible slightly elevated risk of antidepressant use must of course be weighed against impairment of quality of life and established risks of cardiovascular disease and mortality associated with untreated depression.

### Strengths and Limitations of the Study

One major advantage of our study is the completeness of our endpoint-data due to the use of insurance claim data which reduced the limitation of any drop outs. Furthermore, we used population based data which reduces the risk of selection biases. Another advantage comprises the application of a strictly defined clinical endpoint (ischemic stroke) in comparison to other studies which were included in the meta-analyses and partly used intracerebral hemorrhage as well as TIA as outcome measurements [2], [10]. Taking into account the possible variety of stroke risk from different subclasses of antidepressants, one limitation of our study consists of the lack of separate analysis for subclasses of antidepressant medication. Furthermore, in our multivariate model both an elevated GDS-score and the intake of antidepressants were considered as indicators for depressive symptoms, thus it might be difficult to distinguish between the stroke risk of depression itself and the risk caused by antidepressant intake as mentioned. Moreover, the power of our statistical analysis might be diminished if some of the deceased have died from their first ever ischemic stroke without being transferred to hospital.

### Conclusions

To date, there has been no extensive investigation on whether sufficient treatment of depression might influence and potentially improve not only clinically depressive symptoms but also the risk of stroke in depressive patients; this represents a possible target for future prospective studies. Our study suggests that differences exist in the depression-associated stroke risk in elderly sub-groups according to age and sex. Our observations need to be confirmed by further studies. Evidence-based recommendations from randomized clinical trials are still needed to elucidate depression treatment in depressive patients at risk for stroke.

## Supporting Information

Table S1 **Baseline Characteristics of Participants by Age Groups 55–64 and ≥65 Years and GDS <5 and GDS ≥5 or Antidepressants (AD).**
(DOC)
