# Supplementary material for: Depressive Symptoms and the Risk of Ischemic Stroke in the Elderly—Influence of Age and Sex
Source: PLoS One. 2012 Nov 30;7(11):e50803. doi: 10.1371/journal.pone.0050803 (PMC3511268; doi:10.1371/journal.pone.0050803)
Supplement: Table S1 — Baseline Characteristics of Participants by Age Groups 55–64 and ≥65 Years and GDS <5 and GDS ≥5 or Antidepressants (AD). (DOC) [file pone.0050803.s001.doc]

Table S1

|  | All Subjects  N=3852 | Subgroups | | | |
| --- | --- | --- | --- | --- | --- |
|  |  | Age < 65  N=1667(43.3%) | Age ≥ 65  N= 2185 (56.7) | Male  N=1587 (41.2) | Female  N=2265(58.8) |
| Men, n (%) | 1587 (41.2) | 750 (45) | 837 (38.3) | - | - |
| Women (%) | 2265 (58.8) | 917 (55) | 1348 (61.7) | - | - |
| Age, median ±SD | 66.0 ± 7.61 | 60.26 ± 2.71 | 71.00± 5.99 | 65.00 ± 7.09 | 67.00 ±8.12 |
| Age < 65 | 1667 (43.3) | - | - | 750 (47.3) | 917 (40.5) |
| Age ≥ 65 | 2185 (56.7) | - | - | 837 (52.7) | 1348 (59.5)) |
| Antidepressants | 276 (7.2) | 107 (6.4) | 169 (7.7) | 67 (4.2) | 199 (8.8) |
| GDS > 5 | 553 (14.4) | 206 (12.4) | 347 (15.9) | 218 (13.7) | 335 (14.8) |
| GDS>5/Antidepressants | 720 (18.7) | 268 (16.1) | 452 (20.7) | 256 (16.1) | 464 (20.5) |
| Physical activity, n (%) |  |  |  |  |  |
| None | 570 (14.8) | 164 (9.8) | 406 (18.6) | 187 (11.8) | 383 (16.9) |
| Moderate | 1500 (38.9) | 607 (36.4) | 893 (40.9) | 613 (38.6) | 887 (39.2) |
| High | 1778 (46.2) | 894 (53.7) | 884 (40.5) | 784 (49.4) | 994 (43.9) |
| Smoking status, n (%) |  |  |  |  |  |
| Non-smoker | 2519 (65.4) | 1027 (61.6) | 1492 (68.3) | 642 (40.5) | 1877 (82.9) |
| Former smoker | 940 (24.4) | 394 (23.6) | 646 (25.0) | 687 (43.3) | 253 (11.2) |
| Current smoker | 390 (10.1) | 245 (14.7) | 145 (6.6) | 256 (16.1) | 134 (5.9) |
| BMI, mean ±SD | 27.26 ± 4.44 | 27.28 ± 4.55 | 27.27± 4.36 | 27.44 ± 3.84 | 27.10 ± 4.82 |
| Diabetes, n (%) | 775 (20.1) | 249 (14.9) | 526 (24.1) | 369 (23.3) | 406 (17.9) |
| History of stroke n (%) | 130 (3.4) | 33 (2.0) | 97 (4.4) | 66 (4.2) | 64 (2.8) |
| History of TIA, n (%) | 170 (4.4) | 40 (2.4) | 130 (5.9) | 79 (5.0) | 91 (4.0) |
| Hypertension, n (%) | 2209 (57.3) | 779 (46.7) | 1430 (65.4) | 877 (55.3) | 1332 (58.8) |
| History of myocardial infarction, n (%) | 161 (4.2) | 39 (2.3) | 122 (5.6) | 114 (7.2) | 47 (2.1) |
| Atrial fibrillation, n (%) | 181 (4.7) | 33 (2.0) | 148 (6.8) | 92 (5.8) | 89 (3.9) |
| Hyperlipidemia, n (%) | 1670 (43.4) | 667 (40.0) | 1003 (45.9) | 685 (43.2) | 985 (43.5) |
| Antihypertensive medication, n (%) | 2207 (57.3) | 776 (46.6) | 1431 (65.5) | 874 (55.1) | 1333 (58.9) |
| Acetylsalicylacid use, n (%) | 979 (25.4) | 246 (14.8) | 733 (33.5) | 456 (28.7) | 523 (23.1) |
| Statin use n (%) | 619 (16.1) | 223 (13.4) | 396 (18.1) | 287 (18.1) | 332 (14.7) |
| Phenprocoumon use, n (%) | 172 (4.5) | 40 (2.4) | 132 (6.0) | 97 (6.1) | 75 (3.3) |
